# Legal protections for *in vitro* embryos in Paraguay and comparative contexts

**DOI:** 10.3389/frph.2025.1633574

**Published:** 2025-09-05

**Authors:** Evelina Gisela Lezcano, Carlos Gustavo González Morel, Eva Nara Pereira, Danilo Fernández Ríos

**Affiliations:** ^1^ Facultad de Derecho y Ciencias Sociales, Universidad Nacional de Asunción, Asunción, Paraguay; ^2^ Instituto de Investigaciones en Ciencias de la Salud, Universidad Nacional de Asunción, San Lorenzo, Paraguay; ^3^ Facultad de Ciencias Exactas y Naturales, Universidad Nacional de Asunción, San Lorenzo, Paraguay

**Keywords:** assisted reproductive technologies (ARTs), conception, *in vitro* embryos, reproductive rights, right to life

## Abstract

The legal protection of *in vitro* embryos in Paraguay remains unresolved because no specific legislation regulates assisted reproductive technologies. This study analysed Paraguay's constitutional framework, Civil Code, judicial rulings, and international treaties through doctrinal legal analysis, complemented by a comparative review of international regulations and jurisprudence. Findings show that the Paraguayan doctrine and courts consider conception to occur at fertilisation, while the Inter-American Court of Human Rights associates conception with implantation. This difference generates tension between domestic law and international treaty obligations. A comparative analysis with the United Kingdom, Spain, Argentina, and Italy revealed diverse regulatory models and highlighted significant gaps in Paraguayan law concerning embryo storage, research, donation, consent, and dispute resolution. This work provides a comprehensive review of the challenges that Paraguay faces in developing a regulatory framework that balances respect for constitutional principles, alignment with international obligations, and nuanced ethical considerations of human embryos and reproductive rights.

## 1 Introduction

Definitions have a foundational role in both legal and scientific reasoning. In law, definitions determine how facts and conduct are classified under applicable norms. In science, definitions aim for precision by delimiting phenomena based on the current state of knowledge. When legal frameworks seek to regulate scientific development, definitional clarity becomes both a linguistic necessity and a normative act. The case of the human embryo exemplifies this tension, as the term may acquire distinct meanings depending on whether it is invoked by a biologist, ethicist, or judge (1).

The term ‘*in vitro* embryo’ historically refers to embryos that are developed within a laboratory setting using controlled culture conditions (2–4). This approach is integral to various assisted reproductive technologies (ARTs), particularly *in vitro* fertilisation (IVF), which involves the process of fertilising an oocyte with sperm *in vitro*, followed by culturing the resulting embryos before they are transferred to the uterus or cryopreserved for future use (5).

ARTs comprise a range of interventions (6) and are typically categorized into three broad levels. First-level techniques are minimally invasive and involve procedures such as intrauterine insemination, which can be performed with or without prior ovarian stimulation. These methods are commonly used in donor sperm treatment and ovulatory disorders. Second-level techniques are more complex and invasive, involving direct manipulation of male and female gametes, as well as IVF procedures. This category includes IVF with embryo transfer, intracytoplasmic sperm injection, and cryopreservation of gametes and embryos. Clinicians recommend these methods when preimplantation genetic testing is necessary to prevent genetic disorders. Third-level techniques require surgical intervention under general anaesthesia. These procedures involve laparoscopic oocyte retrieval, intra-tubal transfer of gametes, zygotes, or embryos, and microsurgical sampling of sperm directly from the testicles or epididymides (7).

Scientific advancements in ARTs and stem cell research have progressively destabilized the traditional understanding of what constitutes an embryo (8, 9). The concept of the *in vitro* embryo now also includes laboratory-generated structures derived from embryonic or induced pluripotent stem cells (1, 10). Although these stem cell–based embryo models do not result from fertilisation, they reproduce key stages of natural embryonic development. Such developments challenge legal categories rooted in older biological assumptions and raise questions regarding the beginning of life, moral status, and limits of permissible use.

Legal systems have shown varying responses to these challenges. Spain, for example, introduced the concept of the ‘pre-embryo’ (11) and anchored regulatory thresholds in developmental criteria such as the 14-day post-fertilisation rule (12). The European Court of Justice has defined embryos based on their inherent capacity to develop into human beings. Many jurisdictions instead use ambiguous or variably qualified terms such as ‘early human embryo,’ or ‘pre-implantation embryo.’ These definitions rarely correspond directly to scientific classifications; instead, they reflect negotiated compromises between ethical considerations, legal issues, and societal values (1).

ARTs also challenge the traditional understanding of the beginning of human life and personhood. Therefore, the legal field is increasingly confronted with the task of distinguishing between the biological inception of human life and the point at which an *in vitro* embryo is recognised as a person with legal rights. This distinction influences a wide array of considerations about the definition of personhood.^1^ Although extensive literature exists on the ethical considerations of ARTs, research focusing on the legal status and protection of *in vitro* embryos, particularly in Paraguay, remains limited (15, 16).

## 2 Paraguayan legal context

### 2.1 Interpretations of conception

In Paraguay, no specific legislation currently outlines *in vitro* embryos and ARTs (17), leading to a lack of legal protection for embryos that have not yet been implanted (15, 16). This has resulted in a lack of clarity regarding the scope of constitutional rights.

Paraguay's regulatory framework is articulated based on constitutional norms that guarantee the protection of the right to life, reproductive rights, and access to reproductive health plans. International treaties, incorporated into domestic laws, constitute a component of these regulations. The rights and principles enshrined in these instruments serve as guides for protecting life during the early stages of development (Table 1).

**Table 1:** Evolution of Paraguayan legislation related to the protection of the right to life from 1985 to 2017.

Year	Law	Chapter/Article	Provision
1985	Law No. 1183/1985 Paraguayan Civil Code	Title I Natural Persons, Chapter I General Provisions, Art. 28	A natural person has legal capacity from conception to acquire property by donation, inheritance, or bequest. The irrevocability of the acquisition is subordinated to the condition of being born alive, even if only for a few moments after being separated from the mother's womb (99).
1989	Law No. 1/1989 Ratifying the ACHR	Art. 4 The right to life	Every person has the right to have his life respected. This right shall be protected by law and, in general, from the moment of conception. No one shall be arbitrarily deprived of his life (78).
1990	Law No. 57/1990 Approving and ratifying the Convention on the Rights of Children	Art 1	For the purposes of this Convention, a child refers to every human being below the age of 18 years unless, under the law applicable to the child, the majority is attained earlier (100).
1992	Law No. 5/1992 Approving the accession of the Republic to the International Covenant on Civil and Political Rights	Part III Art. 6.1	The right to life is inherent to the human person. This right shall be protected by law. No one shall be arbitrarily deprived of life (101).
1992	Constitution of the Republic of Paraguay	Art. 4 The right to life; Art. 50 Right to form a family; Art. 61 Family planning and maternal and child health	The right to life is guaranteed, in general, from the moment of conception (77); Right to form a family. The State recognises the right of individuals to decide freely and responsibly the number and frequency of the birth of their children, as well as to receive, in coordination with the relevant agencies, education, scientific guidance, and adequate services in this area (77); Reproductive health plans are guaranteed (77).
1993	Decree No. 16078/1993 Accepting the competence of the IACtHR to hear and try human rights cases	Art. 1	Recognition of the jurisdiction of the IACtHR (79).
2001	Law No. 1680/2001 Code for Children and Adolescents	Art. 9 Protection of unborn persons	The protection of unborn children is exercised through the care of pregnant women, from conception to 45 days after birth (102).

The Constitution guarantees the right to life from the moment of conception, as stipulated in Article 4. This protection is reinforced by the incorporation of international treaties ratified by law, including the American Convention on Human Rights (ACHR) (18, 19), International Covenant on Civil and Political Rights,^2^ and Convention on the Rights of the Child.^3^ However, the precise meaning of the term “conception” remains a topic of contemporary debate, particularly regarding its exact beginning, which is crucial for rendering rights effective (22).

The Paraguayan Civil Code acknowledges the inception of a natural person from the moment of conception, albeit without delineating the precise instant at which this occurs. In the absence of any explicit reference to ‘in the mother's womb’ in the Civil Code to denote conception, the domestic legal doctrine has endeavoured to bridge this interpretive gap. Consequently, it can be inferred that human life conceived via IVF is legally safeguarded (23).

It has been argued that the conception of a new human being should be viewed as the moment when fertilisation occurs, either inside or outside the mother's womb, through ARTs in a laboratory (23). This perspective holds that the embryo, whether implanted or not, has personhood and is entitled to legal protection, albeit in a limited and conditional manner, because it must be born alive. Therefore, the destruction of embryos is equivalent to that of human life (24–27).

### 2.2 Judicial ruling in Paraguay

Paraguay's judicial system has been subject to contentious issues. In 2018, an amparo^4^ action was initiated, which led the courts to examine the legal safeguards for the embryo in the case of ‘Amparo promoted by MCGG v PG Director of Clínica G’. The plaintiff's main request was to compel the clinic to continue with IVF treatment, which had been halted because of the male donor's refusal to proceed with the final stage of embryo implantation into the mother's uterus. The Adolescent Criminal Appeals Chamber of the Capital, resolved to grant the protection action, promoted and, consequently, forced the clinic to continue the embryo implantation procedure. The Chamber argued that the National Constitution establishes the right to life from conception, interpreting the term conception as the moment of fertilisation of the oocyte with sperm. Therefore, denying embryo implantation in the mother's womb would be an attack on the right to life (29).

The Chamber conducted an exhaustive analysis of domestic legislation, commencing with the Constitution, which safeguards the right to life from the moment of conception. Subsequently, it referred to the Penal Code, wherein it equates the terms ‘embryo’ and ‘foetus’ for the purpose of protecting the right to life, thereby establishing that anyone causing the death of an embryo would be subject to the criminal offence of abortion.

Additionally, the Court cited the Civil Code and the Code of Childhood and Adolescence, which grant protection to unborn individuals from the time of conception. The Court provided an interpretation of the term ‘conception,’ asserting that it should be defined as the moment of fertilisation, irrespective of whether it occurs within or outside the mother's womb (30). Considering this interpretation, the Court decided that conception had already occurred in the case under examination.

After thoroughly examining the issue of the commencement of the right to life, the Court attended to the claim made by the male donor that his readiness to revoke informed consent for treatment should be considered. In this regard, the Court employed the Estoppel^5^ judicial device to fortify the irrevocability of informed consent, grounded in the fact that the male donor, who agreed to undergo treatment by signing a contract, was obligated to engage in conduct consistent with this commitment.

Finally, the Court decided to grant a protective order and ordered the clinic to proceed with the implantation of the embryos in the petitioner's uterus. This ruling highlights the balance of rights between the right to life on one hand, and the right to privacy and not wanting to be a father, on the other, offering a conclusion that could contrast with the position assumed by Paraguay, specifically regarding that of the Inter-American Court of Human Rights (IACtHR) in the ruling Artavia Murillo vs. Costa Rica.

Regarding the analysis of the doctrine and the preceding ruling, it is evident that conception is linked to fertilisation. This indicates that life begins at the instant the oocyte is fertilised by sperm and is not contingent upon whether this occurs spontaneously or through artificial means in the laboratory. An *in vitro* embryo is categorised as analogous to the classification of unborn individuals in the Paraguayan Civil Code. This designation implies that the embryo is entitled to rights commencing from the moment of conception, under the condition that it is born alive.

## 3 Discussion

### 3.1 Legal and ethical considerations

The legal status of human *in vitro* embryos ranges from being considered a person to being viewed as mere property. The concept of an embryo as a person from conception is debated, with some arguing that life is a continuum from conception to death and that the transition from potential person to person occurs at birth (32).

The perspective that human life commences at the moment of conception, which is characterised by the fusion of male sperm and female oocyte, is a prominent viewpoint in the domain of reproductive rights. This perspective emphasises the emergence of a distinct biological entity with a unique genetic legacy (33) entitled to legal and ethical protection from the moment of conception. This viewpoint significantly influences the legal and political discourse regarding the rights of the embryo and its protection, particularly in debates surrounding ARTs, cellular research, and reproductive rights. The significance of this perspective is evident in its impact on shaping the laws and policies related to the inception of human development.

**Figure 1:**
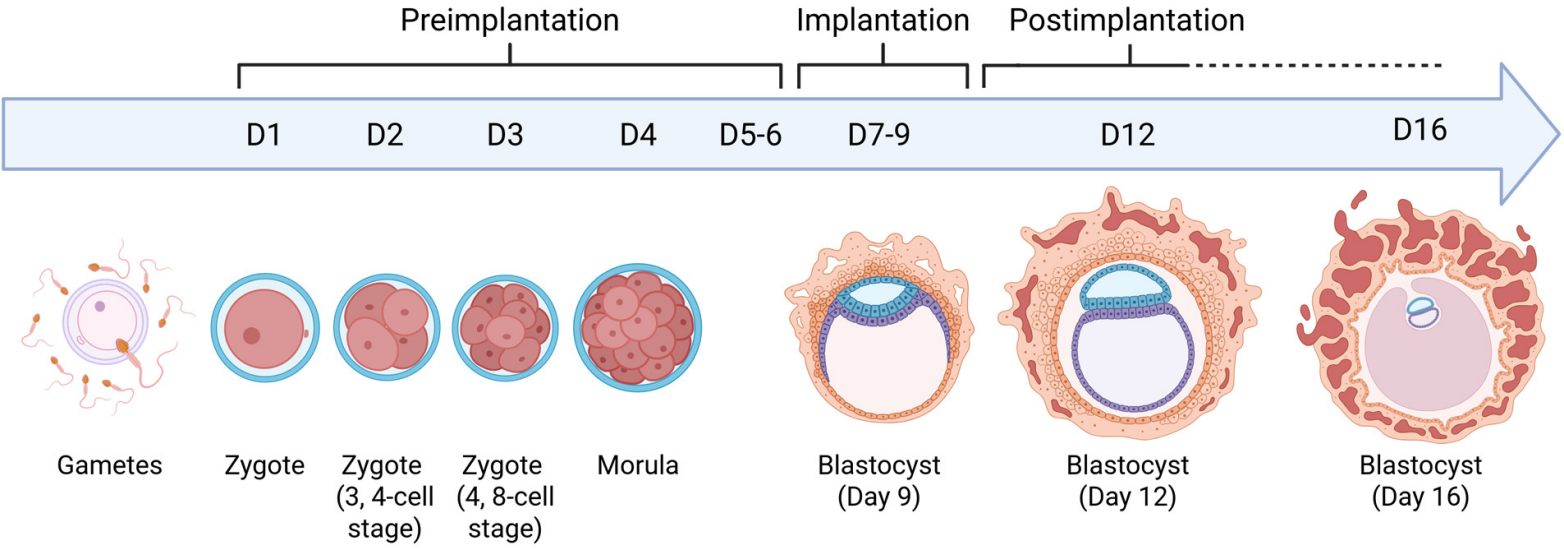
Pre-implantation, implantation, and post-implantation. The gametes fuse during fertilization to form the zygote. The blastocyst forms around day 5. The blastocyst begins to implant and invades the uterine epithelium around day 7. Around day 12, the inner surface of the trophoblast and the outer region of the amniotic ectoderm and yolk sac endoderm are covered by the extraembryonic mesoderm. The figure was created in BioRender. https://app.biorender.com/citation/67f7d37a44eae55a5bad0035.

Another relevant aspect of this discussion is the nesting perspective, which posits that human life commences at the moment of embryo implantation in the uterus, a process that occurs between days 8 and 9 after fertilisation (34) (Figure 1). This stage signifies the onset of pregnancy, which can be identified by the presence of the human chorionic gonadotropin hormone in women (3).

Advocates of the nesting perspective argue that, with nesting, the new individual acquires traits that define uniqueness or the quality of being unique, and individuality or the quality of being one (35). Prior to nesting, an embryo can divide and produce identical individuals (monozygotic twins) (27), and a large number of embryos can be discarded naturally (36). Therefore, there is a certain possibility of development only after implantation, and with this, the embryo becomes a subject of protection. The coexistence of an embryo and its mother is necessary for the embryo to have the elements required for its development and growth; this condition occurs only at the time of implantation (3, 37).

The central nervous system formation offers a unique perspective on this debate, arguing that a pivotal moment in human life is linked to the formation of the primitive nervous system, which is a process that occurs after nesting. This development occurs during the gastrulation phase, when the embryo transforms from a bipartite structure consisting of the epiblast and hypoblast into a three-layer configuration composed of the ectoderm, mesoderm, and endoderm. During this transition, the first signs of nervous system formation are evident (38).

Advocates contend that the inception of cerebral activity signifies the initiation of human existence because the capability of the brain is a distinguishing characteristic of human beings in relation to other life forms. Experts typically concur that this stage occurs approximately 14–15 days after fertilisation (3, 27).

Proponents of granting protection posited that the embryo, with its potential for human life, should have the same status as a child or adult (39). Conversely, critics have argued that since an embryo is composed of cells, it should not be considered a person until implantation in the womb, and thus should not be conferred a special protected status. This reflects the tension between viewing the embryo as potential human life and as a cluster of cells (40).

### 3.2 The 14-day rule

The Warnock Report^6^ (WR) of the United Kingdom serves as a prominent illustration of applying the central nervous system formation in a legal context (41). This report was produced by a panel of experts commissioned to provide recommendations on ART. WR centres on the significant question of the embryo's status, eliciting different viewpoints. The decision to use the formation of the “primitive streak” in the early embryo as a basis for the 14-day rule was a key decision. This rule has been used as a guideline for experimental research in this field for decades (42).

WR declined to address the ontological question of whether an embryo becomes a person or provides a definitive response. Instead, it emphasises the need to treat embryos ethically (43). The report argued that the embryo should be legally protected from day 14 of gestation (14-day period post-fertilisation), which coincides with the emergence of the primitive streak of the nervous system (12). The primary consequence of this conclusion is that scientific research is permissible with embryos that develop up to day 14 of gestation and forbids research on embryos after the 14th day of existence (44, 45).

WR justified the 14-day restriction by presenting both utilitarian and scientific arguments. Utilitarian arguments emphasised the potential advantages of research, whereas scientific arguments were largely influenced by the advice of embryologist Dr. Anne McLaren, who believed that individual human life begins around the time when the appearance of the primitive streak of the brain is observed (46). However, WR recognised that the 14-day limit was an arbitrary compromise designed to alleviate public concerns and provide scientists with the necessary time and conditions for experimentation (43, 47).

### 3.3 International perspectives

Legal systems exhibit significant diversity in their approaches to defining and regulating the legal status of embryos. This diversity is shaped by a combination of scientific, ethical, religious, and socio-political factors that influence both statutory laws and regulatory practices.

Countries such as Austria, Canada, France, Germany, Japan, the Netherlands, and Singapore leave room for interpretation within regulatory guidelines, allowing flexibility in application. This strategy can lead to ambiguity but also enables adaptation as new medical knowledge emerges. Other countries maintain minimal or no formal regulations regarding ARTs, where professional bodies may issue guidelines but lack legislative authority (7, 48).

Australia's ART Act 2024 is being implemented in stages. The primary objective is to enhance public confidence by introducing increased oversight, transparency, and safeguards. The legislation prioritizes the welfare and interests of individuals undergoing ART procedures and emphasizes the well-being of ART-conceived individuals (49).

Switzerland initially had restrictive ART laws prohibiting the culture of more than three zygotes. This led to the transfer of cleavage-stage and multiple embryos to avoid the risk of no embryo being available for transfer on day 5, resulting in high rates of multiple births. In 2017, Swiss ART regulations became more permissive, allowing up to 12 zygotes to be cultured and cryopreserved as blastocysts. This has led to an increase in single and blastocyst embryo transfers, reducing multiple births. Legislative change also caused an increase in both fresh and frozen embryo transfers and a shift from cleavage-stage to blastocyst transfers (50).

In the Philippines, the Reproductive Health Law is among the most controversial pieces of legislation. In a country with a predominantly Catholic population, various religious and conservative groups have questioned its constitutionality and actively sought to delay or block its implementation. While reproductive health care is defined as access to a full range of methods, facilities, services, and supplies that support reproductive health and well-being by addressing related issues, it does not include any mention of ARTs in its provisions (51).

In regions such as Muslim-majority countries, religious doctrines exert a pronounced influence, and ART regulation has been shaped by both legislative actions and fatwas^7^, which serve as guidelines within Sunni Muslim contexts. While these fatwas have guided regulatory developments for subsequent regulations, their dissemination and implementation have varied widely (53).

Understanding Paraguay's legal stance for the protection of *in vitro* embryos requires contextualisation within comparative legislative models. Different jurisdictions have addressed embryo protection through ‘value-oriented,’ ‘procedure-oriented,’ or ‘hybrid’ regulatory approaches (54). A comparative analysis reveals variation in how legal systems protect *in vitro* embryos, allowing for a more precise assessment of Paraguay's position within the global spectrum of reproductive rights legislation. Here, we selected the United Kingdom, Spain, Argentina, and Italy to represent the three regulatory approaches (Table 2).

**Table 2:** Comparative overview of the regulations on assisted reproductive techniques and embryo protection.

Comparative criteria	United Kingdom	Spain	Argentina	Italy
Regulations/ rulings	HFE Act 2008 (103)	Law No. 14/2006 on ART (11)	Constitution, Civil Code, Law No. 26862/2013 (104); Comprehensive access to medical assistance procedures and techniques for reproduction and Regulatory Decree No. 956/2013 (105)	Law 40/2004 Norms in Matters of Medically Assisted Procreation (106)
Biological stage	Primitive streak	Primitive streak	Implantation	Fertilisation
Beginning of human life	Not specified	Not specified	Conception	Not specified
Legal status of the *in vitro* embryo	Not specified	Not specified	To be regulated by a special law (not existing as of 2023)	Not specified
Requirements for access to ARTs	Woman over 18 years of age, independent of sexual orientation and marital status	Woman over 18 years old, Full legal capacity^a^, independent of sexual orientation and marital status	Woman over 18 years old, independent of sexual orientation and marital status	Married or cohabiting adult couples of different sexes, proof of sterility or infertility
Conditions for ARTs	Considers well-being of the unborn child, parental education	Reasonable chances of success, lack of serious risk to the woman's health, implantation of up to 3 embryos per cycle	Not specified	Couples should be offered with the possibility of adoption
Informed consent	Written, revocable prior to implantation	Written, revocable prior to implantation	Written, and free, notarized or certified by the health authority, revocable prior to implantation, renewable for each reproductive cycle	Written, revocable prior to fertilization
Embryo donation	Permitted (nonprofit), financial compensation is allowed	Permitted (nonprofit), financial compensation is allowed	Permitted (nonprofit)	Allowed^b^
Donor anonymity	Identifiable donor	Unidentifiable donor, exception in criminal cases or risk to the child's health or life	Unidentifiable donor, exception with judicial authorization	Not specified
Cryopreservation of embryos	Allowed, 5-year term	Allowed, 2-year term (renewable)	Allowed, time not specified	Not allowed
Destination of surplus embryos	Donation, research, destruction	Donation, research, destruction	Not specified	Not specified
Use of embryos for scientific research	Allowed for embryos up to 14 days of development	Allowed for embryos up to 14 days of development	Limitation of not causing genetic alteration of the embryo	Not allowed, except for therapeutic and diagnostic purposes related to the embryo intended to protect its health and development in the absence of alternative methodologies
Preimplantation genetic diagnosis (PGD)	Permitted, under license from HFEA^c^	Permitted	Not specified	Not allowed

^a^ Legal capacity comprises of two components. The first is the ability to possess rights, which acknowledges the individual as a legal subject with privileges and obligations. The second component is the capacity to act, which enables the individual to engage in legal transactions with other subjects, form and dissolve legal relationships, and seek redress from the relevant authorities. Legal capacity empowers individuals to enter into contracts, marry, vote, have their decisions upheld by law, and other such rights. Consequently, legal capacity signifies the law's acknowledgement of an individual's decision-making capabilities and moral agency, and grants them the status of a person through a legal framework (107).

^b^ In Law 40/2004, heterologous ART was not allowed, and this provision was judicially repealed having been declared unconstitutional by the Italian Constitutional Court (108).

^c^ Human Fertilisation and Embryology Authority.

These approaches differ based on specific legal elements (55, 56): (i) legal status of *in vitro* (non-implanted) embryos, (ii) access to and practice of ARTs, (iii) informed consent requirements, (iv) embryo donation policies, (v) embryo cryopreservation regulations, (vi) disposal of surplus embryos, (vii) use of embryos for scientific research and experimentation, and (viii) pre-implantation genetic diagnosis protocols.

The United Kingdom and Spain apply ‘procedure-oriented’ approaches, influenced by WR and subsequently undergoing legislative reforms (57). Italy exemplifies a ‘value-oriented’ approach, emphasizing the protection of *in vitro* embryos. Argentina, on the other hand, has embraced a regulatory framework that aligns with the ‘hybrid’ approach. However, it remains in its nascent stage and is somewhat limited in scope compared to other jurisdictions.

### 3.4 Rulings of the inter-American court and foreign jurisdictional bodies

The Artavia Murillo v. Costa Rica ruling of 2012 by the IACtHR was a significant legal decision regarding the protection of *in vitro* embryos, considering various perspectives on the beginning of human life and the use of ARTs (Table 3). This case began with Executive Decree 24029-S issued by Costa Rica's Ministry of Health on 3 February 1995 which authorised and regulated the practice of IVF exclusively for married couples (58).

**Table 3:** Comparative analysis of international judicial decisions on the beginning of human life and the protection of *in vitro* embryos.

Jurisdiction/court	Case name and year	Case background	Key principles	Court's decision	Implications for embryo status
Organization of American States (IACtHR)	Artavia Murillo and others vs. State of Costa Rica, 2012	Challenge against the Costa Rican ban on IVF for violating the right to life	The *in vitro* embryo is not a person. The right to life has gradual protection, commencing post-implantation	Found Costa Rica in violation of human rights for banning IVF (58)	Embryo not granted personhood; right to life considered post-implantation
United Kingdom (Grand Chamber of the European Court)	Evans vs. United Kingdom, 2007	Dispute over the use of frozen embryos after the withdrawal of consent by the male partner	The state has the discretion to define when life begins. Embryos lack independent rights under Article 2 of the European Convention on Human Rights	No violation of the European Convention by the UK's decision (73)	Embryos do not have autonomous rights or the right to life
Spain (Constitutional Court)	Ruling No. 53, 1985; No. 212, 1996; No. 116, 1999	Cases dealing with abortion, embryo use, and assisted reproduction laws	The right to life is not absolute; special protection for the *nasciturus*. Distinction between viable and non-viable embryos	Upheld existing laws; no unconstitutionality is found (69–71)	Embryos may be used for research under certain regulated conditions, including obtaining specific informed consent from donors and prior approval of the research project by an ethics committee
Argentina (Supreme Court of Justice)	Portal de Belén Asociación Civil sin Fines de Lucro c. Ministerio de Salud y Acción Social de la Nación s. Amparo, 2002	A case against the marketing of an emergency contraceptive arguing that it violates the right to life by preventing the nesting of a fertilized oocyte	Human life begins at fertilisation; preventing nesting is considered an attack on the right to life	The court granted a writ of amparo, ordering the Ministry of Health to cancel the drug's marketing authorization (63)	The decision affirms the legal recognition of life beginning at fertilisation, impacting the status and rights of embryos
Argentina (National Chamber of Appeals in Civil Matters Room J)	P.A. v. S.A.C. s/ Precautionary Measures, 2011	Dispute over the implantation of cryopreserved embryos, despite the husband's refusal to consent	Informed consent is irrevocable in terms of embryo use and implantation	Granted amparo action, authorizing embryo implantation (65)	Emphasizes the binding nature of informed consent in embryo-related decisions and affirms the status of embryos under this consent
Argentina (National Chamber of Appeals in Civil Matters Room K)	D. P., R. V. v. F., A. E. s/ Medidas Precautorias, 2017	Implantation of cryopreserved embryos following husband's revocation of consent	Informed consent is revocable prior to embryo implantation as per the Civil Code and Law 26832/2013	Did not grant authorization for implantation of cryopreserved embryos (64)	Reinforces the principle that informed consent is pivotal in determining the fate and use of embryos in reproductive treatments
Italy (Constitutional Court)	Rulings N° 151/2009 and 162/2014	Legal challenges against restrictions on IVF techniques, including single embryo implantation and the use of donor gametes	Prohibiting certain IVF practices violated human dignity and reproductive rights	Overturned restrictions on IVF practices, citing constitutional violations (108, 109)	Legal recognition of broader reproductive rights, including choices in IVF practices

Judicial decisions from jurisdictions including the OAS, United Kingdom, Spain, Argentina, and Italy are presented, focusing on the protection of the *in vitro* embryo and legal interpretations of the beginning of human life.

The decree was challenged because it violated the right to life, leading the Supreme Court of Justice of Costa Rica to declare it unconstitutional and ban IVF treatment. This ruling also prohibited ARTs in Costa Rica. Consequently, many couples were forced to suspend treatment or seek treatment abroad.

IACtHR decision in Artavia Murillo v. Costa Rica was significant in protecting women's procreative autonomy and reinforcing an interpretation of the ‘right to life’ that favours procreative autonomy. It revoked a previous decision by the Constitutional Chamber of Costa Rica which banned IVF (59). The ruling emphasised reproductive rights as a human right and placed women's rights over embryo rights. It differentiated between fertilisation and conception, arguing that the legal protection of an embryo from conception does not apply between its creation by fertilisation and implantation *in utero* (6).

Costa Rica was the only country in Latin America that completely banned access to IVF, citing embryos’ right to life. This position was challenged by the IACHR and later by the IACtHR (60). Its influence on other Latin American countries, including laws regarding the beginning of life and ARTs, has been noted (61, 62).

The primary issue with which the IACtHR grapples is the interpretation of Article 4.1 of ACHR, which pertains to the right to life protected from the moment of conception. Article 1.2 of the ACHR defines a person as a human being. The central question is whether *in vitro* embryos can be considered humans (2). The IACtHR went into the interpretation of the terms, ‘conception’ and ‘in general.’ It asserted that given the absence of a consensus at the beginning of life, the term “conception” should be understood as the moment of implantation. Therefore, it is inappropriate to apply Article 4.1 of the ACHR before this event occurs.

The IACtHR highlights that scientific evidence distinguishes between two crucial moments in embryonic development: fertilisation and implantation. Only after the completion of the second event could conception be considered to have occurred. Fertilisation of the oocyte results in a cell with sufficient genetic information for the potential development into a human being, and the lack of implantation into the maternal uterus nullifies the possibility of development.

Regarding the expression, ‘in general,’ the IACtHR maintained that it allows for exceptions to be made to the principle of protecting the right to life from conception. The IACtHR further supported its argument by emphasising that the right to life is not an absolute right that justifies the complete denial of other rights and that the protection of life is gradual and incremental, varying depending on the degree of development.

The Supreme Court of the Nation in Argentina adopted a unique stance on the issue of conception, asserting that it occurs at the moment of fertilisation (63). Consequently, the Court determined that the embryo should be recognised as an individual from that instant onwards. This interpretation adhered to lower court rulings (64, 65) until the implementation of the new Civil Code in 2015 (66) and Law 26862/2013 (67), which established the regulations for access to ARTs.

After the enactment of these regulations, there has been a trend towards granting embryos special protection without assigning rights until implantation. Argentine courts adopted the same interpretation as the IACtHR in Artavia Murillo vs. Costa Rica, granting rights to the embryo from the moment of implantation, and national norms established the revocability of informed consent until the moment of implantation (68).

On the other hand, the Constitutional Court of Spain has examined the constitutionality of legislation pertaining to abortion (69), embryo and foetal donation and use (70), and ARTs (71). In these deliberations, it adhered to a consistent line of interpretation; while denying the unborn child^8^ the ownership of the right to life, it acknowledged that it deserves special protection as a constitutionally protected legal entity. Consequently, the legality of permissible embryo experimentation has been upheld, provided that it is conducted in accordance with human dignity and sanctioned research on embryos under specific conditions.

The United Kingdom's judicial system, as established in Evans v. United Kingdom, does not consider the embryo as a person with rights protected under the Convention for the Protection of Human Rights and Fundamental Freedoms (73). This viewpoint aligns with the notion that legislation should allow couples to undergo IVF treatment and that couples have the power to revoke their consent at any time before embryo implantation. This approach was later supported by the European Court of Human Rights in its ruling on 7 March 2006 which emphasised that the determination of the beginning of the right to life was within the discretionary scope of the Member States.

According to British jurisprudence, an embryo lacks autonomous rights or interests, and is neither capable of claiming, nor has the right to life claimed in its name, in accordance with Article 2 of the European Convention (74). This understanding highlights the absence of a violation of European regulations in this regard, reflecting the intricacy and diversity of approaches to the regulation of reproductive rights and the protection of the embryo in the European legal framework.

In 2009 and 2014, the Italian Constitutional Court declared unconstitutional the norms of Law 40/2004 that provided in ART the obligation to obtain and implant up to three embryos per reproductive cycle and the prohibition of heterologous fertilisation (75, 76).

### 3.5 Future challenges

In Paraguay, the lack of specific regulations on ARTs has created a significant challenge in forming a legal framework that protects the rights of individuals using these technologies. The domestic legal hierarchy places the Constitution of the Republic of Paraguay as the highest authority, followed by the international treaties ratified by law (77).

The Constitution recognises a supranational legal order that guarantees human rights, peace, justice, cooperation, and development in various spheres (77). Within this framework, Paraguay incorporated the ACHR into its domestic law (78) and accepted the contentious jurisdiction of the IACtHR (79).

The IACtHR introduced the ‘doctrine of conventionality control’ in its rulings, beginning with Myrna Mack Chang v. Guatemala (80–82), and further developed in Almonacid Arellano v. Chile (83) and Trabajadores Cesados del Congreso v. Peru (84). This doctrine allows judges in signatory states to compare domestic laws with international treaty obligations, requiring them to prioritise treaty norms when conflicts arise. In cases where a domestic norm contradicts an international provision, judges may set aside national laws to uphold treaty obligations.

The IACtHR emphasised that upon ratifying an international treaty like the ACHR, states must ensure that domestic laws do not undermine the treaty's dispositions. This involves interpreting national laws in line with the ACHR, as interpreted by the IACtHR, which serves as the ultimate authority on the Convention's meaning (83).

In Paraguay, there are divergent views on applying conventionality control, particularly when a constitutional provision appears to be incompatible with ACHR or IACtHR interpretations. Proponents argue that Paraguay's Constitution endorses a supranational order, requiring alignment with international treaties as per the Vienna Convention ratified by Law No. 289 (85), mandating compliance with treaty obligations. This interpretation supports the modification^9^ of the Constitution to confer constitutional status on international human rights treaties (86).

Critics of conventionality control argue that it lacks normative support within domestic law, where the Constitution is supreme, and assert that international courts should not override constitutional primacy (87). Additionally, conventionality control may pose practical challenges for local judges, who lack the authority to disregard national laws in favour of international ones (87, 88).

In ‘Amparo promoted by MCGG v PG Director of Clínica G ‘ (29), the Paraguayan courts declined to apply conventionality control, adhering strictly to domestic laws rather than following the IACtHR guidelines in Artavia Murillo v. Costa Rica regarding the right to life.

Both, the ACHR and Paraguay's Constitution, broadly recognise the protection of the right to life from conception, yet the definition of ‘conception’ varies, affecting the scope of legal protection. According to the interpretation of domestic courts and the local doctrine, conception is defined as the moment of fertilisation, conferring personhood status on *in vitro* embryos, and indicating a value-oriented approach.

Adopting the interpretive standards set in Artavia Murillo v. Costa Rica, Paraguay could consider conception to occur at implantation, potentially allowing for ART procedures during the pre-implantation stages. This highlights the challenge of balancing embryo personhood with the use of ARTs.

Comprehensive guidelines for embryo storage, research, and donation are necessary to facilitate the use of ARTs. Domestic laws should also address consent, filiation, and civil liability in cases of embryonic harm or loss to ensure the protection of all parties involved. A particular challenge in regulating ARTs involves the development of legal processes to resolve conflicts between the rights of ART users and *in vitro* embryos.

The only legal case concerning ARTs in Paraguay involved an amparo action. This type of action does not result in a final decision, allowing for further litigation with expanded terms and evidence. To handle ART-related disputes effectively, procedural law should evolve to provide mechanisms for timely and thorough judicial responses, promote access to justice for all parties, and ensure comprehensive consideration of evidence and arguments.

Even as Paraguay continues to address local legal and ethical hurdles in regulating ARTs, certain emerging technological challenges demand attention. To navigate through these evolving issues, Paraguay must foster national debate involving politicians, judges, medical professionals, bioethicists, and researchers. Such dialogue is critical for developing adaptive frameworks that balance ethical considerations, constitutional principles, and implications of scientific progress.

The proposal to extend the culture period of human embryos in the laboratory from 14 to 28 days has the potential to significantly reshape our understanding of human development (41, 89) and necessitate a reassessment of the moral value attributed to embryos during the earliest stages (90).

Genome-editing technologies represent a dual-edged sword in embryonic research. While these tools hold immense clinical promise, they also introduce putative risks, such as off-target effects, which require careful oversight (91).

Additionally, the development of human embryo models derived from stem cells, which can mimic stages beyond the 14-day post-implantation period (92), blurs the lines between naturally occurring and laboratory-produced embryos, creating a regulatory gray area.

The advent of unisexual reproduction has raised various legal considerations,^10^ and the possibility of producing nonviable embryos using synthetic DNA^11^ technology has raised more questions about the moral status of human embryos.

Breakthroughs in artificial womb technology, initially designed to improve outcomes in premature infants (95, 96), open new possibilities but also provoke debates about parental rights and the legal status of entities developing within these systems. The terminology used to describe these entities has also come under scrutiny and some researchers argue that terms like ‘neonate’ are inappropriate due to their implication of live birth, leading to the proposal of alternatives, such as ‘foetonates’ or ‘foetal neonates’ (97).

These discussions require clear definitions and updated legal frameworks to address possible implications. It is essential to conduct a comprehensive regulatory review to balance potential benefits with respect to human dignity (98).

## 4 Recommendations

This analysis of the legal protection afforded to *in vitro* embryos in Paraguay reveals several key findings and areas for future consideration. Paraguay currently lacks comprehensive legislation to regulate ARTs and the legal status of *in vitro* embryos. This regulatory gap creates uncertainty regarding the rights and protection of embryos created by ARTs.

Paraguay's Constitution and ratified international treaties provide general protection for the right to life from conception. However, the precise definition and implications of ‘conception’ in the context of *in vitro* embryos remain subject to interpretation.

The limited case law in Paraguay tends to interpret conception as occurring during fertilisation, granting personhood status to *in vitro* embryos. This value-oriented approach contrasts with more permissive interpretations of other jurisdictions. Paraguay's domestic interpretation of embryo rights diverge from the guidelines established by the IACtHR. This creates potential conflict between domestic and international legal obligations.

Paraguay needs comprehensive and specific legislation on ARTs, covering all aspects related to the subject, including who can benefit from these treatments, which techniques are permitted or prohibited, and other relevant issues. There is a pressing need for informed public discourse to shape Paraguay's approach to such complex questions.

Significant work remains to be performed to develop a comprehensive, ethically sound, and scientifically informed regulatory system for *in vitro* embryos and ARTs. As reproductive technologies continue to advance, Paraguay faces the challenge of crafting legislation that respects constitutional principles, aligns with international obligations, and addresses the nuanced ethical considerations surrounding human embryos and reproductive rights.

## Footnotes

^1^Historically, personhood has been defined by attributes such as consciousness, self-awareness, and free will, all of which are subject to debate. The ACHR recognises the right to life and includes a consensus definition that personhood begins with conception. This concept is fundamental to human rights concerns, as reflected in the first draft of the ACHR (13, 14).

^2^The International Covenant on Civil and Political Rights acknowledges the need for special protection for unborn children, as indicated by its provisions prohibiting the execution of pregnant women. This implies the recognition of an unborn child as a human being that requires protection (20).

^3^The Convention on the Rights of the Child, while not explicitly stating the protection of life from conception, asserts that a child requires special safeguards and care, including appropriate legal protection before and after birth (21). This suggests the acknowledgment of the rights of the unborn child, although the extent and nature of these rights are subject to interpretation.

^4^In most legal systems in the Spanish-speaking world, the writ of *amparo*, also known as ‘*recurso de amparo’* or ‘*juicio de amparo,*’ serves as a remedy for the protection of constitutional rights (28).

^5^Estoppel is an equitable doctrine, a bar that prevents one from asserting a claim or right that contradicts what one has said or done before, or what has been legally established as true (31).

^6^The WR, published in 1984, forms the basis of the UK legislation on embryo research. This was the first report of its kind to consider regulation of advancements in human fertilization and embryology. The report was developed after extensive consultation and consideration of various ethical viewpoints and evidence submitted to the Warnock Committee (39).

^7^Fatwas represent formal legal opinions or interpretations in Islamic jurisprudence, traditionally issued by recognized religious scholars to address queries concerning Islamic law and its application (52).

^8^The Constitutional Court of Spain employs the term ‘nasciturus’ in its rationale, yet it does not specify the particular stage of human development that the term encompasses. However, nasciturus can be understood to refer to the ‘conceived but not yet born’ based on the precedents set by Roman law, which have had a significant influence on Spanish law (72).

^9^The Constitution of Paraguay provides two procedures for modification: amendment and reform. Depending on which provisions are modified, this may give rise to another debate on whether the provision on the hierarchy of laws can be modified by reform or amendment.

^10^The recent generation of bi-paternal mice via targeted imprinting modifications and their potential to enhance embryonic stem cell development further complicate the debate on embryo protection (93).

^11^Refers to the artificial synthesis of DNA sequences with the aim of reconstructing the genomes of various organisms. This technology has the potential to generate a human genome devoid of genes essential for embryonic development. Such a genome could then be introduced into oocyte that are in the early stages of development but lack the capability to progress to full-term development (94).

## References

[1] Piciocchi C Martinelli L. The change of definitions in a multidisciplinary landscape: the case of human embryo and pre-embryo identification. Croat Med J. (2016) 57(5):510–5. 10.3325/cmj.2016.57.510 27815942 PMC5141455

[2] Lamm E. El status del embrión in vitro y su impacto en las técnicas de reproducción humana asistida. Aclarando conceptos para garantizar derechos humanos. La Ley Suplemento Especial Nuevo Código Civil y Comercial de la Nación. Familia: Filiación y Responsabilidad Parental (2015) 43. p. 1–23. Available online at: https://colectivoderechofamilia.com/el-status-del-embrion-in-vitro-y-su-impacto-en-las-tecnicas-de-reproduccion-humana-asistida-aclarando-conceptos-para-garantizar-derechos-humanos/ (Accessed August 20, 2023).

[3] Goldsztern de Rempel NE. Una breve referencia a la reproducción humana desde la biología y la medicina. In: La Protección Jurídica del Embrión. Ciudad Autónoma de Buenos Aires: Ediar (2016). p. 29–54.

[4] Araújo J Carvalho AS Silvestre M Martinho Da Silva P. Embryo. In: Ten Have H, editor. Encyclopedia of Global Bioethics. Cham: Springer International Publishing (2016). p. 1095–105. 10.1007/978-3-319-09483-0_169

[5] Ragab ARA. Infertility. In: Ten Have H, editor. Encyclopedia of Global Bioethics. Cham: Springer International Publishing (2016). p. 1610–9. 10.1007/978-3-319-09483-0_243

[6] Zegers-Hochschild F Dickens BM Dughman-Manzur S. Human rights to *in vitro* fertilization. Int J Gynaecol Obstet. (2013) 123(1):86–9. 10.1016/j.ijgo.2013.07.001 23932062

[7] Matteo M. Assisted reproductive technology. In: Bettocchi C Busetto GM Carrieri G Cormio L, editors. Practical Clinical Andrology. Cham: Springer International Publishing (2023). p. 237–50. (Accessed August 4, 2025). 10.1007/978-3-031-11701-5_18

[8] De Miguel Beriain I. What is a human embryo? A new piece in the bioethics puzzle. Croat Med J. (2014) 55(6):669–71. 10.3325/cmj.2014.55.669 25559839 PMC4295065

[9] Martinelli L Busatta L Galvagni L Piciocchi C. Social egg freezing: a reproductive chance or smoke and mirrors? Croat Med J. (2015) 56(4):387–91. 10.3325/cmj.2015.56.387 26321034 PMC4576754

[10] M’hamdi HI. Language and labels from the lab: definitions in the stem cell-based embryo model debate. Stem Cell Rep. (2025) 20(5):102477. 10.1016/j.stemcr.2025.102477 PMC12143142 40250440

[11] Jefatura del Estado. Ley 14/2006, de 26 de mayo, sobre técnicas de reproducción humana asistida. Sect. 1, Ley 14/2006 May 27. (2006). p. 19947–56. https://www.boe.es/eli/es/l/2006/05/26/14 (Accessed November 11, 2023).

[12] Blackshaw BP Rodger D. Why we should not extend the 14-day rule. J Med Ethics. (2021) 47(10):712–4. 10.1136/medethics-2021-107317 34112713 PMC8479730

[13] Herrmann JR. Legal hybrids: on the legal Status of embryos and foetuses. Med Law Int. (2009) 10(1):1–22. 10.1177/096853320901000101

[14] Johnson MH Grudzinskas G Cohen J. Personhood: to be or when to be – is that the question? Reprod Biomed Online. (2012) 24(7):687–8. 10.1016/j.rbmo.2012.04.008 22710733

[15] de Riquelme FJD Rojas AFA Figueredo AMM. Regulación de las técnicas de reproducción humana asistida por el ordenamiento jurídico paraguayo. Revista Internacional de Investigación en Gobernabilidad. (2024) 4(1):33–44.

[16] Ortiz MM. El estatuto jurídico del embrión humano en la fecundación *in vitro*. La Saeta Universitaria Académica y de Investigación. (2020) 9(1):1–8. 10.56067/saetauniversitaria.v9i1.209

[17] Torres G Shapiro A Mackey TK. A review of surrogate motherhood regulation in South American countries: pointing to a need for an international legal framework. BMC Pregnancy Childbirth. (2019) 19(1):46. 10.1186/s12884-019-2182-1 30691390 PMC6350392

[18] Joseph R. Children’s rights ‘without any exceptions whatsoever’. In: Human Rights and the Unborn Child. Leiden: Brill (2009). p. 283–300. 10.1163/ej.9789004175600.i-350

[19] Fuentes A Vannelli M. Expanding the protection of children’s rights towards a dignified life: the emerging jurisprudential developments in the Americas. Laws. (2021) 10(4):84. 10.3390/laws10040084

[20] Joseph R. American Convention on human rights: ‘in general, from the moment of conception’. In: Human Rights and the Unborn Child. Leiden: Brill (2009). p. 213–44. 10.1163/ej.9789004175600.i-350.190

[21] Onuegbulam MC. Comparative analysis of the legal Status of an unborn child in criminal law in Nigeria, America, United Kingdom (UK), and India. ABUAD Law Journal. (2021) 9(1):51–71. 10.53982/alj.2021.0901.04-j

[22] Ruppel E. Abortion in Paraguay Case Study. Augustana Center for the Study of Ethics Essay Contest (2016). p. 1–11. Available online at: https://digitalcommons.augustana.edu/ethicscontest/8/

[23] Moreno Ruffinelli JA. Capítulo IV la persona. In: Riveros Arce G, editor. Derecho Civil Parte General Personas. 7th edn Asunción: Intercontinental Editora (2005). p. 159–82.

[24] Escurra Paez CE. Doctrina. La aplicación de las técnicas de reprodución humana asistida. (TRHA). la fecundación *in vitro* (FIV). el consentimiento informado. Derecho a la identidad e información. Breve exposición. In: Gaceta Judicial. Asunción: Intercontinental Editora (2021). p. 19–40.

[25] Martínez Núñez I. Temas de Bioética. Nuestra Experiencia. Asunción: Ediciones El Lector (2017).

[26] Moreno Ruffinelli JA. Disposiciones generales. Artículo 28. In: Código Civil de la República del Paraguay Comentado. 2nd Ed Asunción: La Ley Paraguaya (2011). p. 3–22.

[27] Sapena Giménez J. Estatus jurídico del embrión *in vitro*. Implicancias jurídicas y de otros órdenes de los descubrimientos científicos y tecnológicos en el área de la medicina, la genética y las ciencias de la vida. In: Bioética, Derechos Humanos y Derecho de Familia. Asunción: Intercontinental Editora (2013). p. 298–314.

[28] Brewer-Carías AR. Judicial review and amparo proceedings in Latin America. In: Brewer-Carías AR, editor. Constitutional Protection of Human Rights in Latin America: A Comparative Study of Amparo Proceedings. Cambridge: Cambridge University Press (2008). p. 87–91. 10.1017/CBO9780511551727.006

[29] Tribunal de Apelaciones de la Niñez y Adolescencia de la capital. Jurisprudencia. Constitución de la república del Paraguay. Acción de amparo. Juzgado de primera instancia de la niñez y la adolescencia 08/06/2018 (S.D. No 13). In: Schlichting Garcete E Green Da Re S, editors. Gaceta Judicial. Asunción: Instituto de Investigaciones Jurídicas e Intercontinental Editora (2021). p. 47–52.

[30] Cruz-Coke R. Fundamentos genéticos del comienzo de la vida humana. Revista Chilena de Pediatría. (1980) 51(2):121–4. 10.4067/S0370-41061980000200006 6893496

[31] Cornell Law School. *estoppel*. LII/Legal Information Institute (2022). Available online at: https://www.law.cornell.edu/wex/estoppel (Accessed January 23, 2024)

[32] Raposo VL Osuna E. Embryo dignity: the status and juridical protection of the *in vitro* embryo. Med Law. (2007) 26(4):737–46. 18284114

[33] Morales Godo J. Ciencia, Ética y Derecho (A propósito de la inseminación artificial y la fecundación extrauterina). Docentia et Investigatio. (2012) 14(1):31–47. https://revistasinvestigacion.unmsm.edu.pe/index.php/derecho/article/view/10165 (Accessed October 29, 2023).

[34] Longo LD. Embryology and early developmental physiology. In: The Rise of Fetal and Neonatal Physiology. New York: Springer New York (2013). p. 113–35. 10.1007/978-1-4614-7921-5_7

[35] Obladen M. Animatio: ideas about the beginning of personhood. In: Obladen M, editor. Oxford Textbook of the Newborn. Oxford: Oxford University Press (2021). p. 3–10. 10.1093/med/9780198854807.003.0001

[36] Sharkey AM Macklon NS. The science of implantation emerges blinking into the light. Reprod Biomed Online. (2013) 27(5):453–60. 10.1016/j.rbmo.2013.08.005 24055396

[37] Kurjak A. Controversies on the beginning of human life – science and religion closer and closer. Science Art Religion. (2022) 1(1–2):23–45. 10.5005/sar-1-1-2-23 34010252

[38] López-Moratalla N. El cigoto de nuestra especie es cuerpo humano. Persona Bioética. (2010) 14(2):120–40.

[39] Hammond-Browning N. Ethics, embryos, and evidence: a look back at warnock. Med Law Rev. (2015) 23(4):588–619. 10.1093/medlaw/fwv028 26232721

[40] Deitch R. Government looks for guidance through warnock minefield. Lancet. (1984) 324(8411):1107–8. 10.1016/S0140-6736(84)91550-2 11644294

[41] Appleby JB Bredenoord AL. Should the 14-day rule for embryo research become the 28-day rule? EMBO Mol Med. (2018) 10(9):e9437. 10.15252/emmm.201809437 30087137 PMC6127884

[42] Franklin S. Developmental landmarks and the warnock report: a sociological account of biological translation. Comp Stud Soc Hist. (2019) 61(04):743–73. 10.1017/S0010417519000252

[43] Colomer MF Pastor LM. The preembryós short lifetime. The history of a word. Cuadernos Bioética. (2012) 23(3):677–94. 23320640

[44] Hurlbut JB Hyun I Levine AD Lovell-Badge R Lunshof JE Matthews KRW Revisiting the warnock rule. Nat Biotechnol. (2017) 35(11):1029–42. 10.1038/nbt.4015 29121021

[45] Raposo VL. The new Japanese regulation on human/non-human chimeras: should we worry? JBRA Assist Reprod. (2021) 25(1):155–61. 10.5935/1518-0557.20200045 33118717 PMC7863089

[46] Ferrer Colomer M Pastor LM. Génesis y uso del término ‘pre embrión’ en la literatura científica actual. Persona Bioética. (1998) 2:2–27.

[47] Perazzo G Gargiulo L. Informe warnock: revisión y reflexión bioética a los 25 años de su publicación. Vida Ética. (2019) 10(1):8–25.

[48] Ginoza MEC Isasi R. Regulating preimplantation genetic testing across the world: a comparison of international policy and ethical perspectives. Cold Spring Harbor Perspect Med. (2020) 10(5):a036681. 10.1101/cshperspect.a036681 PMC7197420 31506325

[49] Australian Capital Territory. Assisted reproductive technology act 2024 (2025). Available online at: https://www.legislation.act.gov.au/a/2024-7/ (Accessed August 7, 2025)

[50] Pape J Levy J Makieva S Von Wolff M. Legal framework and IVF outcomes: a comparative analysis of fresh and frozen embryo transfers in Switzerland. Reprod Biomed Online. (2025) 50(2):104483. 10.1016/j.rbmo.2024.104483 39754834

[51] Biana HT. The need for assisted reproductive technology regulations: a case for women in the Philippines. Front Sociol. (2025) 10:1429980. 10.3389/fsoc.2025.1429980 40182237 PMC11966037

[52] Whyte S. Are fatwas dispensable? Examining the contemporary relevance and authority of fatwas in Australia. Oxford Journal of Law and Religion. (2023) 11(2–3):314–42. 10.1093/ojlr/rwac015

[53] Kooli C. Review of assisted reproduction techniques, laws, and regulations in Muslim countries. Middle East Fertil Soc J. (2020) 24(1):8. 10.1186/s43043-019-0011-0

[54] Penasa S. Converging by procedures: assisted reproductive technology regulation within the European Union. Med Law Int. (2012) 12(3–4):300–27. 10.1177/0968533213485749

[55] Raposo VL. Assisted reproduction two models of regulation: Portugal v. Spain. J Brasil Reprod Assist. (2012) 16(1):35–43.

[56] Bolaños Guevara A. Aspectos a considerar en la legislación comparada de la fecundación asistida: argentina, españa e italia. Revista Parlamentaria. (2014) 21(1):85–117.

[57] Cutting R. Legislation in the United Kingdom. In: Nagy ZP Varghese AC Agarwal A, editors. Practical Manual of in Vitro Fertilization. Advanced Methods and Novel Devices. New York: Springer New York (2012). p. 605–9. 10.1007/978-1-4419-1780-5_68

[58] Corte Interamericana de Derechos Humanos. Artavia Murillo y Otros (‘Fecundación in Vitro’) vs. Costa Rica. San José: Corte Interamericana de Derechos Humanos (2012). Available online at: https://www.corteidh.or.cr/docs/casos/articulos/seriec_257_esp.pdf (Accessed October 28, 2023).

[59] Pires TT. Procreative autonomy, gender equality and right to life: the decision of the inter-American court of human rights in artavia murillo v. Costa Rica. Rev Direito GV. (2017) 13(3):1007–28. 10.1590/2317-6172201739

[60] Hevia M Herrera Vacaflor C. *The legal status of in vitro fertilization in latin America and the American convention on human rights*. Suffolk University Law School (2012). Available online at: https://papers.ssrn.com/abstract=2160733 (Accessed January 25, 2023).

[61] Hevia M. The legal Status of surrogacy in Latin America. In: Rivera-López E Hevia M, editors. Controversies in Latin American Bioethics. Cham: Springer International Publishing (2019). p. 53–63. 10.1007/978-3-030-17963-2_4

[62] Mateos CI. Influencia de la CorIDH en el derecho interno. Comienzo de la vida y reproducción humana asistida en relación con nuestro nuevo código civil y comercial. Argument Estudios Transdiscipl Sobre Cult Jurídicas Admin Justicia. (2017) 4:46–60. 10.26612/2525-0469/2017.4.03

[63] Corte Suprema de Justicia de la Nación. Portal de Belén -Asociación Civil sin Fines de Lucro c. Ministerio de Salud y Acción Social de la Nación s. Amparo [Internet]. (2002). Available online at: http://www.saij.gob.ar/corte-suprema-justicia-nacion-federal-ciudad-autonoma-buenos-aires-portal-belen-asociacion-civil-sin-fines-lucro-ministerio-salud-accion-social-nacion-amparo-fa02000003-2002-03-05/123456789-300-0002-0ots-eupmocsollaf?q=moreLikeThis%28id-infojus%2C%20numero-norma%5E4%2C%20tipo-documento%5E4%2C%20titulo%5E4%2C%20jurisdiccion%2C%20tesauro%2C%20provincia%2C%20tribunal%2C%20organismo%2C%20autor%2C%20texto%5E0.5%29%3Aportal%20de%20belen&o=4&f=Total%7CFecha/2002%5B20%2C1%5D%7CEstado%20de%20Vigencia%5B5%2C1%5D%7CTema%5B5%2C1%5D%7COrganismo%5B5%2C1%5D%7CAutor%5B5%2C1%5D%7CJurisdicci%F3n%5B5%2C1%5D%7CTribunal/CORTE%20SUPREMA%20DE%20JUSTICIA%20DE%20LA%20NACION%7CPublicaci%F3n%5B5%2C1%5D%7CColecci%F3n%20tem%E1tica%5B5%2C1%5D%7CTipo%20de%20Documento/Jurisprudencia&t=6 (Accessed November 19, 2023).

[64] Cámara Nacional de Apelaciones en lo Civil. D. P., R. V. c. F., A. E. s. Medidas Precautorias. Capital Federal, Ciudad Autónoma de Buenos Aires (2017). Available online at: https://www.saij.gob.ar/camara-nacional-apelaciones-civil-nacional-ciudad-autonoma-buenos-aires—medidas-precautorias-fa11020046-2011-09-13/123456789-640-0201-1ots-eupmocsollaf? (Accessed November 02, 2023).

[65] Cámara Nacional de Apelaciones en lo Civil. P., A. c. S., A. C. s. Medidas Precautorias. Capital Federal, Ciudad Autónoma de Buenos Aires (2011). Available online at: https://www.saij.gob.ar/camara-nacional-apelaciones-civil-nacional-ciudad-autonoma-buenos-aires—medidas-precautorias-fa11020046-2011-09-13/123456789-640-0201-1ots-eupmocsollaf? (Accessed November 02, 2023).

[66] Código Civil y Comercial de la Nación. Buenos Aires: Ministerio de Justicia y Derechos Humanos de la Nación (2015). Available online at: http://www.bibliotecadigital.gob.ar/items/show/2690 (Accessed November 3, 2023)

[67] Reproducción médicamente asistida. *Ley N° 26862 - reglamentación*. (2013). Available online at: https://www.argentina.gob.ar/normativa/nacional/decreto-956-2013-217628 (Accessed November 11, 2023)

[68] Herrera M. Argumentos jurídicos a favor de la postura según la cual el embrión *in vitro* o no implantado no es persona humana. Reproducción. (2015) 30(4):161–3.

[69] Pleno del Tribunal Constitucional. *Recurso previo de inconstitucionalidad N° 800/1983 contra el texto definitivo del proyecto de ley orgánica de reforma del art. 417 bis del código penal. 53/1985* (1985). Available online at: https://hj.tribunalconstitucional.es/es-ES/Resolucion/Show/433#complete_resolucion&completa (Accessed November 02, 2023).

[70] Pleno del Tribunal Constitucional. Sentencia 212/1996, de 19 de diciembre de 1996. Recurso de inconstitucionalidad 596/1989. Promovido por 79 Diputados del Grupo Parlamentario Popular contra la Ley 42/1988. (1997). Available online at: https://www.boe.es/buscar/doc.php?id=BOE-T-1997-1180 (Accessed November 02, 2023).

[71] Pleno del Tribunal Constitucional. Recurso de inconstitucionalidad número 376/1989, promovido por don Federico Trillo-Figueroa Martínez-Conde, Comisionado por 63 Diputados, contra la Ley 35/1988, de 22 de noviembre. 1989. Available online at: https://www.boe.es/buscar/doc.php?id=BOE-A-1989-6631 (Accessed November 18, 2023).

[72] Larrea Simball LJ Tutiven Abad TC. The right to life of the unborn child from a human rights perspective. Centro Sur. (2023) 7(2):1–17. 10.37955/cs.v7i2.308

[73] Cabezudo Bajo MJ. *Gran sala: evans vs. reino unido (demanda no 6339/05). Sentencia. Abogacía general del estado, dirección del servicio jurídico del estado; subdirección general de constitucional y derechos humanos; Área de derechos humanos* (2007). Available online at: https://hudoc.echr.coe.int/app/conversion/pdf/?library=ECHR&id=001-201170&filename=CASE%20OF%20EVANS%20v.%20THE%20UNITED%20KINGDOM%20-%20%5BSpanish%20Translation%5D%20by%20the%20Spanish%20Ministry%20of%20Justice.pdf (Accessed November 02, 2023).

[74] Ben-Naftali O Canor I. Evans v. United Kingdom. American Journal of International Law. (2008) 102(1):128–34. 10.1017/S0002930000039889

[75] Benagiano G Filippi V Sgargi S Gianaroli L. Italian Constitutional court removes the prohibition on gamete donation in Italy. Reprod Biomed Online. (2014) 29(6):662–4. 10.1016/j.rbmo.2014.08.013 25311973

[76] Benagiano G Gianaroli L. The Italian constitutional court modifies Italian legislation on assisted reproduction technology. Reprod Biomed Online. (2010) 20(3):398–402. 10.1016/j.rbmo.2009.11.025 20093085

[77] Convención Nacional Constituyente. Constitución de la República del Paraguay. Asunción: Gaceta Oficial (1992). Available online at: https://www.csj.gov.py/cache/lederes/G-63-22061992-0.pdf (Accessed November 01, 2023).

[78] Poder Legislativo del Paraguay. *Ley 1/1989 que aprueba y ratifica la convención americana de derechos humanos o pacto de san josé de costa rica*. Asunción (1989). Available online at: https://www.csj.gov.py/cache/lederes/P-1-081989-L-1-1.pdf (Accessed November 01, 2023).

[79] Presidencia de la República del Paraguay. *Decreto 16078/1993 por el cual se acepta la competencia de la corte interamericana de derechos humanos para el conocimiento y juzgamiento de violaciones a los derechos humanos*. Asunción: Decree (1993). Available online at: https://www.csj.gov.py/cache/lederes/R-2-011993-D-16078.pdf (Accessed November 18, 2023).

[80] Carbonell Sánchez M. Introducción general al control de convencionalidad. In: González Pérez LR Valadés D, editors. El Constitucionalismo Contemporáneo: Homenaje a Jorge Carpizo. México: Universidad Nacional Autónoma de México (2013). p. 67–95. Available online at: https://repositorio.unam.mx/contenidos/5009444

[81] Corte Interamericana de Derechos Humanos. Caso Myrna Mack Chang Vs. Guatemala. San José: Fondo, Reparaciones y Costas (2003). Available online at: https://www.corteidh.or.cr/ver_ficha_tecnica.cfm?nId_Ficha=287&lang=en (Accessed May 1, 2025)

[82] Hitters JC. Control de convencionalidad (adelantos y retrocesos). Estud Const. (2015) 13(1):123–62. 10.4067/S0718-52002015000100005

[83] Corte Interamericana de Derechos Humanos. Caso Almonacid Arellano y Otros Vs. Chile. San José: Excepciones Preliminares, Fondo, Reparaciones y Costas (2006). Available online at: https://www.corteidh.or.cr/ver_ficha_tecnica.cfm?nId_Ficha=335&lang=en (Accessed May 1, 2025)

[84] Corte Interamericana de Derechos Humanos. Caso Trabajadores Cesados del Congreso (Aguado Alfaro y Otros) Vs. Perú. San José: Excepciones Preliminares, Fondo, Reparaciones y Costas (2006). Available online at: https://www.corteidh.or.cr/ver_ficha_tecnica.cfm?nId_Ficha=192&lang=en (Accessed May 1, 2025)

[85] Poder Legislativo del Paraguay. Ley 289/1971 Que Aprueba la Convención de Viena Sobre el Derecho de los Tratados. Asunción (1971).

[86] Giménez de Allen ME. Control de convencionalidad: herramienta de uso obligatorio para los jueces. Rev Jurídica UCA Law Rev. (2016) 25:787–800.

[87] Moreno Rodríguez D. Una mirada escéptica al control de convencionalidad. Rev Jurídica UCA Law Rev. (2016) 25:1071–8.

[88] Acosta Insfrán MI. Control difuso de convencionalidad a la luz del control de constitucionalidad en Paraguay. In: Comentario a la Constitución, Tomo V, editor. Homenaje al Vigésimo Quinto Aniversario. Asunción: Corte Suprema de Justicia, Instituto de Investigaciones Jurídicas (2018). p. 17–58. Available online at: https://www.pj.gov.py/ebook/libros_files/Comentario_a_la_Constitucion_Tomo_V.pdf (Accessed August 31, 2024).

[89] The next debate on embryo science. Nat Biotechnol. (2021) 39(8):895–895. 10.1038/s41587-021-01032-0 34341556

[90] Peters T. Keep the 14-day rule in stem cell research. Theol Sci. (2021) 19(3):177–83. 10.1080/14746700.2021.1944484

[91] Riveros Maidana R Méndez Ferreira A Benítez Candia N Nara Pereira E Fernández Ríos D. Sistema CRISPR/cas: edición genómica de precisión. Mem Inst Investig Cienc Salud. (2020) 18(1):97–107. 10.18004/mem.iics/1812-9528/2020.018.01.97-107

[92] Oldak B Wildschutz E Bondarenko V Comar MY Zhao C Aguilera-Castrejon A Complete human day 14 post-implantation embryo models from naive ES cells. Nature. (2023) 622(7983):562–73. 10.1038/s41586-023-06604-5 37673118 PMC10584686

[93] Li ZK Wang LB Wang LY Sun XH Ren ZH Ma SN Adult bi-paternal offspring generated through direct modification of imprinted genes in mammals. Cell Stem Cell. (2025) 32(3):361–74.e6. 10.1016/j.stem.2025.01.005 39879989

[94] Villalba A Smajdor A Brassington I Cutas D. Non-viable embryos created with synthetic DNA. Trends Biotechnol. (2025):S0167779925000848. 10.1016/j.tibtech.2025.02.021 40133161

[95] Flake AW De Bie FR Munson DA Feudtner C. The artificial placenta and EXTEND technologies: one of these things is not like the other. J Perinatol. (2023) 43(11):1343–8. 10.1038/s41372-023-01716-2 37393398

[96] Kukora SK Mychaliska GB Weiss EM. Ethical challenges in first-in-human trials of the artificial placenta and artificial womb: not all technologies are created equally, ethically. J Perinatol. (2023) 43(11):1337–42. 10.1038/s41372-023-01713-5 37400494

[97] Kozlov M. Human trials of artificial wombs could start soon. Here’s what you need to know. Nature. (2023) 621(7979):458–60. 10.1038/d41586-023-02901-137709976

[98] Peng Y Lv J Xiao Z Ding L Zhou Q. A framework for the responsible reform of the 14-day rule in human embryo research. Protein Cell. (2022) 13(8):552–8. 10.1007/s13238-022-00907-5 35165849 PMC9232680

[99] Poder Legislativo del Paraguay. Ley 1183/1985 Código Civil Paraguayo. Asunción (1985). Available online at: https://www.csj.gov.py/cache/lederes/EDICI%C3%93N%20OFICIAL%20C%C3%B3digo%20Civil%20Paraguayo.pdf (Accessed November 18, 2023).

[100] Poder Legislativo del Paraguay. Ley 57/1990 Que Aprueba y Ratifica la Convención Sobre los Derechos del Niño. Asunción (1990). Available online at: https://www.csj.gov.py/cache/lederes/P-0-20121990-L-57-1.pdf (Accessed November 19, 2023).

[101] Poder Legislativo del Paraguay. Ley 5/1992 que Aprueba la Adhesión de la República al Pacto Internacional de Derechos Civiles y Políticos. Asunción (1992). Available online at: https://www.csj.gov.py/cache/lederes/G-37-09041992-L-5-0.pdf (Accessed November 01, 2023).

[102] Poder Legislativo del Paraguay. Ley 1680/2001 Código de la Niñez y la Adolescencia. Asunción (2001). Available online at: https://www.csj.gov.py/cache/lederes/G-105-04062001-L-1680-1.pdf (Accessed November 19, 2023).

[103] *Human fertilisation and embryology act 2008*. Statute Law Database (2008). Available online at: https://www.legislation.gov.uk/ukpga/2008/22/contents (Accessed November 11, 2023)

[104] Honorable Congreso de la Nación Argentina. *Ley 26862 acceso integral a los procedimientos y técnicas médico asistenciales de reproducción médicamente asistida* (2013). Available online at: https://www.argentina.gob.ar/normativa/nacional/ley-26862-216700 (Accessed November 01, 2023).

[105] Presidencia de la Nación Argentina. Decreto 956 que Reglamenta la Ley No 26.862. *Acceso integral a los procedimientos y técnicas médico-asistenciales de reproducción medicamente asistida*. Decree (2013). Available online at: https://www.argentina.gob.ar/normativa/nacional/decreto-956-2013-217628 (Accessed November 01, 2023).

[106] Parlamento Italiano. *Legge 40/2004 norme in materia di procreazione medicalmente assistita* (2004). Available online at: https://www.parlamento.it/parlam/leggi/04040l.htm (Accessed November 01, 2023).

[107] Arstein-Kerslake A. An empowering dependency: exploring support for the exercise of legal capacity. Scandinavian Journal of Disability Research. (2016) 18(1):77–92. 10.1080/15017419.2014.941926

[108] Corte Costituzionale della Repubblica Italiana. *Sentenza N° 162/2014 giudizio di legitimitá costituzionale in via incidentale* (2014). p. 162. Available online at: https://www.cortecostituzionale.it/documenti/download/doc/recent_judgments/162-2014_en.pdf (Accessed November 19, 2023).

[109] Corte Costituzionale della Repubblica Italiana. *Sentenza no 151/2009 giudizio di legitimitá costituzionale in via incidentale* (2009). p. 151. Available online at: https://www.cortecostituzionale.it/documenti/download/doc/recent_judgments/CC_SS_151_2009_EN.pdf (Accessed November 19, 2023).

